# Proteolysis-Dependent Remodeling of the Tubulin Homolog FtsZ at the Division Septum in *Escherichia coli*

**DOI:** 10.1371/journal.pone.0170505

**Published:** 2017-01-23

**Authors:** Marissa G. Viola, Christopher J. LaBreck, Joseph Conti, Jodi L. Camberg

**Affiliations:** Department of Cell and Molecular Biology, The University of Rhode Island, Kingston, Rhode Island, United States of America; Centre National de la Recherche Scientifique, Aix-Marseille Université, FRANCE

## Abstract

During bacterial cell division a dynamic protein structure called the Z-ring assembles at the septum. The major protein in the Z-ring in *Escherichia coli* is FtsZ, a tubulin homolog that polymerizes with GTP. FtsZ is degraded by the two-component ATP-dependent protease ClpXP. Two regions of FtsZ, located outside of the polymerization domain in the unstructured linker and at the C-terminus, are important for specific recognition and degradation by ClpXP. We engineered a synthetic substrate containing green fluorescent protein (Gfp) fused to an extended FtsZ C-terminal tail (residues 317–383), including the unstructured linker and the C-terminal conserved region, but not the polymerization domain, and showed that it is sufficient to target a non-native substrate for degradation in vitro. To determine if FtsZ degradation regulates Z-ring assembly during division, we expressed a full length Gfp-FtsZ fusion protein in wild type and *clp* deficient strains and monitored fluorescent Z-rings. In cells deleted for *clpX* or *clpP*, or cells expressing protease-defective mutant protein ClpP(S97A), Z-rings appear normal; however, after photobleaching a region of the Z-ring, fluorescence recovers ~70% more slowly in cells without functional ClpXP than in wild type cells. Gfp-FtsZ(R379E), which is defective for degradation by ClpXP, also assembles into Z-rings that recover fluorescence ~2-fold more slowly than Z-rings containing Gfp-FtsZ. In vitro, ClpXP cooperatively degrades and disassembles FtsZ polymers. These results demonstrate that ClpXP is a regulator of Z-ring dynamics and that the regulation is proteolysis-dependent. Our results further show that FtsZ-interacting proteins in *E*. *coli* fine-tune Z-ring dynamics.

## Introduction

Cell division in bacteria is a conserved and highly coordinated dynamic process involving many cellular proteins that function together to divide a single cell into two daughter cells [1]. During cell division the Z-ring assembles at midcell, the site of septation. The Z-ring contains the essential cell division protein FtsZ and many other division proteins, which are recruited to the septum. FtsZ is a GTPase that is structurally homologous to eukaryotic tubulin and forms large, dynamic polymers [2]. Each FtsZ monomer contains a compact, globular N-terminal polymerization domain, a flexible unstructured linker region, and a conserved region near the C-terminus that is important for protein interactions [2,3]. Several proteins in *E*. *coli* bind to FtsZ and have been shown to modulate the polymerization state of FtsZ in vitro, including MinC, SlmA and Z-ring associated proteins (ZAPs) [4]. Many of these protein-protein interactions occur near the FtsZ C-terminus.

High-resolution microscopy of division septa in several organisms, including *E*. *coli*, *Bacillus subtilis*, *Staphylococcus aureus*, *Caulobacter crescentus* and *Streptococcus pneumoniae*, showed that the Z-ring contains a network of overlapping FtsZ polymers staggered around the inner face of the cytoplasmic membrane [5–10]. By fluorescence microscopy, the Z-ring is also frequently observed as a loose helical structure rather than a closed ring. The Z-ring is highly dynamic and rapidly exchanges FtsZ subunits with a reported half-time of approximately 9 sec in *E*. *coli* [5,11–13]. GTP hydrolysis modulates the polymerization state of FtsZ and is thought to be the major factor that promotes dynamic exchange in the Z-ring. An *E*. *coli* strain containing the substitution mutation G105S in FtsZ impairs GTP hydrolysis in vitro and confers a temperature-sensitive growth phenotype [14–16]. Cells containing chromosomal *ftsZ(G105S)*, also referred to as *ftsZ84*, have Z-rings that exhibit 3-fold slower dynamics, suggesting that Z-ring dynamics are coupled to GTP hydrolysis [12]. It has also been reported that cell division proteins, specifically those that interact directly with FtsZ, may modulate Z-ring dynamics in *E*. *coli*. FtsZ-interacting proteins ZapA and ZapB were shown to stabilize the network of overlapping FtsZ polymers in Z-rings in vivo and promote polymer bundling in vitro [17–19].

Counteracting the functions of proteins that stabilize or bundle FtsZ polymers, several proteins antagonize FtsZ polymers and promote their disassembly. In *E*. *coli*, MinC, SlmA, ClpXP and most recently ZapE have been reported to destabilize FtsZ polymers in vitro by promoting disassembly, preventing reassembly or by shifting the equilibrium of FtsZ polymers towards disassembly [4,20–26]. Deletion of the *minCDE* operon from *E*. *coli* leads to the assembly of Z-rings with ~2-fold slower dynamics [12]. The Min system in *E*. *coli*, which includes MinC, MinD and MinE, inhibits Z-ring assembly at non-septal locations by establishing an oscillating polar gradient of MinC, an FtsZ polymerization inhibitor [27]. The contributions of SlmA, which prevents polymerization of FtsZ over the nucleoid, and ZapE, which may destabilize FtsZ polymers during late constriction, to Z-ring dynamics are unknown.

Proteolysis is an important regulatory mechanism for the cell division pathway in bacteria. ClpXP is an ATP-dependent protease that contains the ATP-dependent chaperone unfoldase, ClpX, bound to a compartmentalized protease, ClpP [28]. In *C*. *crescentus*, ClpXP controls initiation of DNA replication through degradation of CtrA and is regulated through phosphoregulated adaptor complexes [29]. In addition, ClpXP degrades FtsZ in non-replicative swarmer cells [30].

In *E*. *coli* ClpXP is known to degrade many diverse substrates [31,32]. ClpXP specifically recognizes, unfolds, and degrades FtsZ in vivo and in vitro, and overexpression of ClpXP leads to cell filamentation and increased degradation of FtsZ [24]. ClpXP degrades approximately 15% of total FtsZ per cell cycle in *E*. *coli* [24]. Furthermore, the ClpX chaperones from *E*. *coli*, *B*. *subtilis*, and *Mycobacterium tuberculosis* have also been shown to antagonize FtsZ polymerization in the absence of ClpP [33–36].

ClpX is a member of the AAA+ superfamily of ATPases and forms a hexameric ring with a central substrate translocation channel. Several eukaryotic AAA+ ATPases, such as spastin and katanin, have been suggested to sever microtubules and alter tubulin dynamics in vivo [37–41]. Spastin and katanin are members of a meiotic clade of AAA+ ATPases, which, along with Vps4, may disassemble polymers [40]. ClpX from *E*. *coli* promotes disassembly and degradation of FtsZ polymers along with its cognate protease ClpP [24]. Two regions of FtsZ are important for recognition and degradation by ClpXP. One region is present in the unstructured linker (residues 352–358) of FtsZ and the other is present near the C-terminus (residues 379–383) [25]. Although FtsZ monomers and polymers are degraded by ClpXP, FtsZ polymers, containing either GTP or the GTP analog GMPCPP, are degraded more efficiently [24,25,42].

Here, we demonstrate that the C-terminal extended tail of FtsZ, which includes the unstructured linker and conserved C-terminal region, but not the polymerization domain, is sufficient to target a non-native substrate for degradation by ClpXP in vitro. To uncover the functional role of FtsZ proteolysis by ClpXP during division, we expressed N-terminal Gfp-FtsZ fusion proteins in cells and monitored fluorescence recovery after bleaching Z-rings in vivo. We show that cells unable to make functional ClpXP, or cells that express an FtsZ mutant protein defective for degradation, have Z-rings that recover fluorescence more slowly than Z-rings in wild type cells. We further show that in vitro, degradation of FtsZ polymers occurs cooperatively, indicating that polymerization of FtsZ promotes recognition by ClpX. Together, these results demonstrate that the role for ClpXP during the division cycle of *E*. *coli* is to engage the FtsZ C-terminal region, degrade FtsZ and regulate FtsZ polymer dynamics.

## Results

### Targeting and degradation of a synthetic fluorescent substrate by addition of the FtsZ extended C-terminus

**Fig 1:**
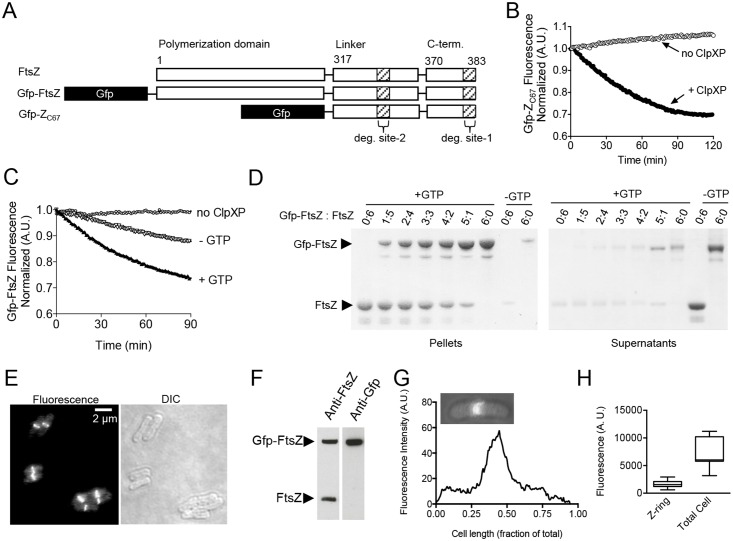
Degradation and localization of Gfp-tagged FtsZ chimeras. (A) Schematic of native FtsZ, Gfp-FtsZ, and Gfp-Z_C67_ showing position of Gfp and FtsZ polymerization domain (1–316), unstructured linker (317–369) and C-terminal (370–383) regions. Sites important for ClpXP degradation are shown (degradation site-1, 379–383; degradation site-2, 352–358). (B) Gfp-Z_C67_ (3 μM) degradation was measured by monitoring loss of fluorescence with time in the absence (white circles) and presence (black circles) of ClpXP (1 μM), ATP (5 mM) and an ATP regenerating system. The curves shown are representative of at least three replicates. (C) Gfp-FtsZ (5 μM) degradation was measured by monitoring loss of fluorescence with time in the presence of ClpXP (1 μM), ATP (5 mM) and a regenerating system in the presence (black triangles) or absence (white triangles) of GTP (2 mM), where indicated. Gfp-FtsZ (5 μM) fluorescence was also measured in the absence of ClpXP (white circles). The curves shown are representative of at least three replicates. (D) Sedimentation of FtsZ (10 μM) and Gfp-FtsZ (10 μM) polymers with GTP, or using different ratios of Gfp-FtsZ to FtsZ (total of 10 μM per reaction), collected by ultracentrifugation. Pellet fractions containing FtsZ polymers and soluble fractions containing non-polymerized FtsZ are shown. (E) Fluorescence microscopy and DIC images of wild type MG1655-derived cells (JC0390) in log phase expressing Gfp-FtsZ induced with 70 μM arabinose. (F) Expression of plasmid encoded Gfp-FtsZ and chromosome encoded FtsZ from cell extracts (1 μg total protein) described in E using antibodies to FtsZ and Gfp. (G) Fluorescence intensity across the long axis of the cell was measured and plotted to determine the relative position of the Z-ring. Inset shows the fluorescence image used for quantitation. Individual cells were chosen as representative of the population. (H) Box and whiskers plot showing total fluorescence at the Z-ring and total cell fluorescence for wild type cells (JC0390) expressing Gfp-FtsZ (n = 11). The extent of the box encompasses the interquartile range of the fluorescence intensity, whiskers extend to the maximum and minimum fluorescence intensities, and the line within each box represents the median.

Recognition and degradation of FtsZ by ClpXP was previously shown by our group to utilize two regions of FtsZ, residues 352–358 in the unstructured linker, and C-terminal residues 379–383 [25]. To determine if an extended C-terminal tail of FtsZ, including the unstructured linker (residues 317–369), the conserved region adjacent to the FtsZ C-terminus (residues 370–379) and the C-terminal variable region (residues 380–383) is sufficient to target a synthetic substrate for degradation, we constructed and purified a fluorescent fusion protein containing Gfp and the extended FtsZ C-terminal tail (Gfp-Z_C67_) (Fig 1A). This chimera does not contain the FtsZ polymerization domain. We measured degradation of Gfp-Z_C67_ in reactions containing ClpXP and ATP by monitoring loss of fluorescence during incubation with ClpXP, indicating that ClpXP unfolds and degrades the Gfp moiety (Fig 1B). After 60 min, approximately 25% of the initial fluorescence was lost under the conditions tested, and then the degradation reaction plateaued (Fig 1B). Gfp without Z_C67_ is stable and not degraded during incubation with ClpXP since it does not contain a ClpX-recognition region (S1A Fig). We also observed that a Gfp-fusion protein containing full-length FtsZ is degraded by ClpXP (Fig 1A and 1C). The loss of fluorescence is attributable to degradation since incubation of Gfp-Z_C67_ or Gfp-FtsZ with ClpX alone, without ClpP, has no effect on the fluorescence of either substrate (S1B Fig). The addition of GTP, which is known to induce FtsZ polymerization, to the degradation reaction containing Gfp-FtsZ increases the rate of degradation from 0.008 units min^-1^ to 0.016 units min^-1^ (Fig 1C). This is in agreement with previous reports showing a 2-3-fold increase in degradation efficiency for native FtsZ in the presence of GTP and suggests that Gfp-FtsZ is capable of polymerization with GTP [24,25]. To confirm this, we performed sedimentation assays using mixtures of FtsZ and Gfp-FtsZ. We observed Gfp-FtsZ sedimentation in the presence of GTP alone and when increasing amounts of FtsZ are included in the reaction, indicating that Gfp-FtsZ polymerizes with GTP (Fig 1D).

### Z-ring localization of Gfp-FtsZ in dividing cells and slow dynamics in *clp* deletion strains

Next, we wanted to determine if Z-ring assembly and dynamics are perturbed in cells lacking ClpXP. To localize fluorescent Z-rings in live, dividing cells, we expressed Gfp-FtsZ from a plasmid in a wild type *E*. *coli* MG1655 strain (JC0390), and in strains deleted for *clpX* (JC0394) or *clpP* (MV0210) (Table 1). Importantly, we used the N-terminal Gfp-FtsZ fusion protein, which is competent for polymerization (Fig 1D) and leaves the C-terminal ClpX interaction site of FtsZ accessible. All strains also constitutively express an arabinose transporter (P_CP18_-*araE*) to reduce cell-to-cell variability in the presence of inducer (arabinose) [43].

**Table 1:** *E*. *coli* strains and plasmids.

Strain or Plasmid	Genotype	Source, reference or Construction^a^^,^^b^^,^^c^
**Strains**		
MG1655	*LAM- rph-1*	[44]
BW27750	*lacI*^q^*rrnB3* Δ*lacZ4787 hsdR514 Δ(araBAD)567 Δ(rhaBAD)568 Δ(araFGH*) *ϕ(ΔaraEp*::*kan* P_CP18_-*araE*)	[43]
BW27784	*lacI*^q^*rrnB3* Δ*lacZ4787 hsdR514 Δ(araBAD)567 Δ(rhaBAD)568 Δ(araFGH*) *ΔaraEp-532*::*frt ϕ* P_CP18_*-araE533*	[43]
JC0390	MG1655 *(ΔaraEp*::*kan* P_CP18_*-araE)*	P1(BW27750) x MG1655
JC0291	MG1655 *ΔclpX*::*frt*	JC0259 [45]; pCP20
JC0394	MG1655 *ΔclpX*::*frt*, *ΔaraEp*::*kan* P_CP18_*-araE*	P1(BW27750) x JC0291
MV03720	MG1655 *ΔclpX*::*kan-parE*	pKD267^c^; λRed
MV03721	MG1655 *clpX-restored*	MV03720; λRed
MV03722	MG1655 *clpX-restored*, *ΔaraEp*::*kan* P_CP18_*-araE*	P1(BW27750) x MV03721
MV0050	MG1655 *ΔclpP*::*frt*	JC0263 [45]; pCP20
MV0210	MG1655 *ΔclpP*::*frt*, *ΔaraEp*::*kan* P_CP18_*-araE*	P1(BW27750) x MV0050
MV0242	MG1655 *ΔclpP*::*kan-parE*	pKD267^c^; λRed
MV0251	MG1655 *clpP(S97A)*	MV0242; λRed
MV0256	MG1655 *clpP(S97A)*, *ΔaraE*::*kan* P_CP18_*-araE*	P1(BW27750) x MV0251
MV03711	MG1655 *clpP-restored*	MV0242; λRed
MV03712	MG1655 *clpP-restored*, *ΔaraEp*::*kan* P_CP18_*-araE*	P1(BW27750) x MV03711
JC0395	MG1655 *ΔminC*::*frt*, *ΔaraEp*::*kan* P_CP18_*-araE*	P1(BW27750) x JC0232 [45]
MV0196	MG1655 *ΔslmA*::*frt*	P1(JW5641) [46] x MG1655; pCP20
MV03730	MG1655 *ΔminC*::*kan-parE*	pKD267^c^; λRed
MV03731	MG1655 *minC-restored*	MV03730; λRed
MV03732	MG1655 *minC-restored*, *ΔaraEp*::*kan* P_CP18_*-araE*	P1(BW27750) x MV03731
MV0198	MG1655 *ΔslmA*::*frt*, *ΔaraEp*::*kan* P_CP18_*-araE*	P1(BW27750) x MV0196
MV0340	MG1655 *ΔzapE*::*frt*	P1(JW3201) [46] x MG1655; pCP20
MV0277	MG1655 *ΔzapE*::*frt*, *ΔaraEp*::*kan* P_CP18_*-araE*	P1(BW27750) x MV0340
MC181	BW27784 λCH151 [P_*lac*_::*zipA-gfp*]	λCH151 [47]
MV0226	BW27784 *ΔclpX*::*kan* λCH151[P_*lac*_::*zipA-gfp*]	P1(JC0259) [45] x MC181
**Plasmids**		
pET-H_6_-Gfp-FtsZ	*kan*	This study
pET-H_6_-Gfp-Z_C67_	*kan*	This study
pET-H_6_-Gfp-Z_C67_(R379E)	*kan*	This study
pCP20	*amp flp* recombinase	[48]
pKD267	*kan* P_*rham*_-*parE*	B. Wanner^c^
pET-ClpP	*kan*	[24]
pET-ClpP(S97A)	*kan*	This study
pET-FtsZ	*kan*	[24]
pET-FtsZ(G105S)	*kan*	This study
pET-FtsZ(R379E)	*kan*	[25]
pET-FtsZ(G105S, R379E)	*kan*	This study
pET-FtsZ(352_7A_)	*kan*	This study
pET-ClpX	*kan*	[24]
pGfp-FtsZ	*amp* P_*ara*_::*gfp-ftsZ*	[45]
pGfp-FtsZ(G105S)	*amp* P_*ara*_::*gfp-ftsZ(G105S)*	This study
pGfp-FtsZ(R379E)	*amp* P_*ara*_::*gfp-ftsZ(R379E)*	[25]
pGfp-FtsZ(G105S, R379E)	*amp* P_*ara*_::*gfp-ftsZ(G105S*, *R379E)*	This study
pGfp-FtsZ(352_7A_)	*amp* P_*ara*_::*gfp-ftsZ(352*_*7A*_*)*	This study

^*a*^ Strain constructions by P1 transduction are described as the following: P1(donor) x recipient.

^*b*^ Where indicated gene deletion donor strains were derived from the Keio Collection [46].

^*c*^ J. Teramoto, K. A. Datsenko, and B. L. Wanner, unpublished results.

**Fig 2:**
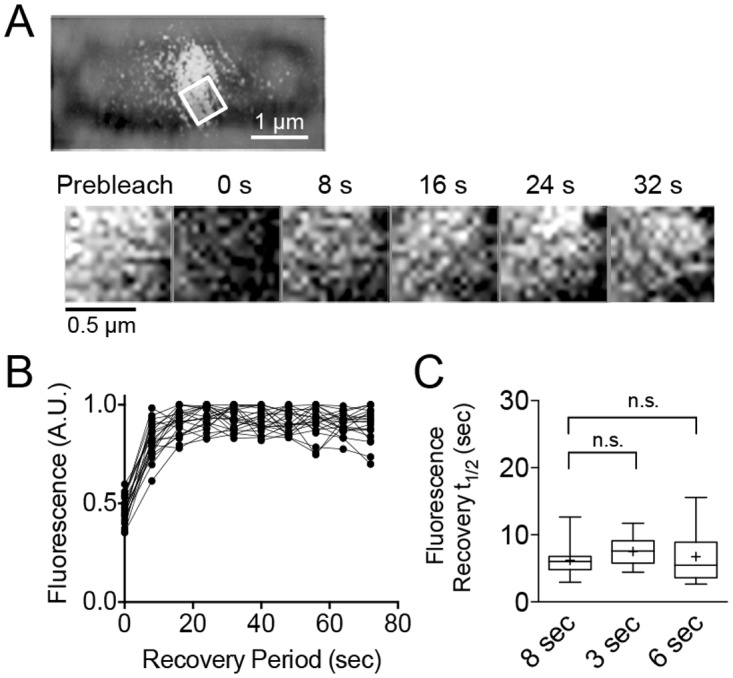
Photobleaching and recovery of the Z-ring in wild type strain. (A) A region of the Z-ring was selected and bleached from wild type cells (JC0390) expressing Gfp-FtsZ and grown in LB containing arabinose (70 μM) as described. Fluorescence recovery in the selected region was monitored every 8 sec for 72 sec (B) and plotted with time. (C) Box and whiskers plot of recovery half-times for Z-rings in wild type cells with various recovery intervals (8 sec, 3 sec, and 6 sec) (n ≥ 16). The extent of the box encompasses the interquartile range of the fluorescence recovery half-times, and whiskers extend to the maximum and minimum values. The line within each box represents the median, with the mean value indicated by ‘+’.

Expression of Gfp-FtsZ in the wild type strain (JC0390) does not interfere with Z-ring assembly or division under the expression conditions tested and constitutes ~50% of the total FtsZ in the cell (Fig 1E and 1F). As expected, fluorescent Z-rings were present at the center of the long axis of dividing cells and the rings contained approximately 20–30% of the total cellular fluorescence (Fig 1G and 1H). To probe the dynamic exchange of fluorescent subunits in the Z-ring of individual dividing cells, we selected a small region of the fluorescent Z-ring in each cell and performed a photobleaching and recovery assay (Fig 2A). After bleaching the fluorescence from the region, recovery in the selected area was monitored at 3, 6 or 8 sec intervals over the next 72 sec, as Gfp-FtsZ from within the Z-ring is exchanged with subunits from the cytoplasm (Fig 2A, 2B and 2C). During the recovery period, we measured the fluorescence at each interval for each region, calculated the recovery half-time, and then determined the average recovery half-time for all replicates. The fluorescence recovery half-time of Z-rings in wild type, dividing cells expressing Gfp-FtsZ is 6.2 ± 0.5 sec (n = 18, 8 sec interval) (Fig 2C) (S1 Table), which is faster but within error of half-times reported previously (9.0 ± 3 and 8.3 ± 3 sec for wild type Z-rings in *E*. *coli* and *B*. *subtilis* respectively) [12]. Recoveries measured at shorter capture intervals (6 sec and 3 sec) generated average half-time values similar to the value measured using an 8 sec interval (6.8 ± 0.5 sec and 7.5 ± 0.5 sec, respectively) (Fig 2C).

**Fig 3:**
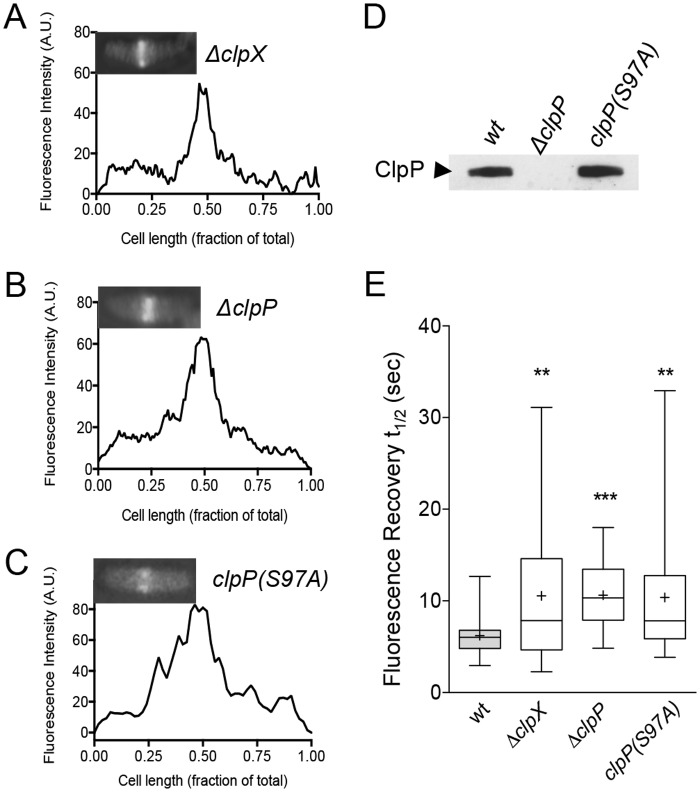
Z-ring assembly and dynamics in *clp* deficient strains. Fluorescence intensity across the long axis of the cell was measured and plotted for cells deleted for *clpX* (JC0394) (A), *clpP* (MV0210) (B), and cells with chromosomal *clpP(S97A)* (MV0256) (C). Insets show the fluorescence image used for quantitation. Individual cells were chosen as representative of the population. (D) Expression of ClpP and ClpP(S97A) from wild type (JC0390), *ΔclpP* (MV0210), and *clpP(S97A)* (MV0256) cell extracts (40 μg total protein) using antibodies to ClpP. (E) Box and whiskers plot of recovery half-times for Z-rings in wild type cells (JC0390) and cells deleted for *clpX* (JC0394), *clpP* (MV0210), and containing chromosomal *clpP(S97A)* (MV0256). The extent of the box encompasses the interquartile range of the fluorescence recovery half-times, and whiskers extend to the maximum and minimum values. The line within each box represents the median, with the mean value indicated by ‘+’. Where indicated, *p* values are specified as ‘**’ (*p*<0.01) or ‘***’ (*p*<0.001) as compared to wild type.

Next, we expressed Gfp-FtsZ in cells deleted for *clpX* or *clpP*, and observed fluorescent Z-rings at midcell, and cells appeared similar to wild type cells expressing Gfp-FtsZ (Figs 1G, 3A and 3B) (S2A Fig). We also constructed a strain in which the chromosomal *clpP* gene was replaced with a gene at the native locus encoding protease-defective *clpP(S97A)* and visualized Z-rings in ClpP-defective cells by expression of Gfp-FtsZ (Fig 3C and 3D) (S2A Fig) [49,50]. We observed no apparent cell division or morphological defects in the strain expressing ClpP(S97A) and Z-rings appeared similar to Z-rings in wild type and *clpP* deletion strains (Fig 1G) (S2A Fig). Next, we selected and bleached regions of fluorescent Z-rings in *clp* deficient cells and monitored fluorescence recovery. Surprisingly, in all *clp* deficient strains, we observed an average recovery half-time significantly longer by 70% than the half-time observed in the parental strain (average recovery half-time of the Z-ring is 10.6 ± 1.3 sec in the *ΔclpX* strain, 10.6 ± 0.7 sec in the *ΔclpP* strain, and 10.4 ± 0.4 sec in the *clpP(S97A)* strain) (Fig 3E) (S2B, S2C and S2D Fig) (S1 Table). We also observed that the distributions of values are larger in *clp* deficient strains, suggesting that there is more heterogeneity among the Z-rings, which could indicate that there is less control over regulating Z-ring architecture in a given population of cells. Finally, we restored wild type copies of *clpX* and *clpP* to *clp* deletion strains at their native loci by recombination, expressed Gfp-FtsZ from a plasmid in the restored strain and performed bleaching and recovery assays of fluorescent Z-rings to determine if we could rescue the slow average recovery half-time (S2A, S2E, S2F, S2G and S2H Fig) (Table 1). We observed that strains restored with *clpX* (MV03722) or *clpP* (MV03712) at the native locus have average half-time recoveries of 8.2 ± 1.4 sec and 8.1 ± 0.6 sec, respectively. Although the values are not significantly different from the value observed for the wild type parental strain (JC0390), it is interesting to note that the strain restored with *clpX* has a slightly lower level of ClpX expression than the wild type strain, which could explain the modest increase in recovery half-time compared to the wild type strain (S1 Table).

Deletion of *clpX* or *clpP* prevents degradation of FtsZ in vivo, leading to slower FtsZ protein turnover as measured in antibiotic chase assays [24]. To test if slow Z-ring fluorescence recovery could be attributable to higher levels of Gfp-FtsZ, we directly tested if a higher expression level of Gfp-FtsZ also leads to slower recovery, but did not observe a significant difference in recovery half-time at a higher arabinose concentration (7.5 ± 0.8 sec) (S3A and S3B Fig). This is in agreement with a previous report showing that an increase in FtsZ expression does not alter the architecture of the Z-ring [5]. These results show that Z-rings in cells lacking functional ClpXP have slow recovery after bleaching and are less dynamic in early division.

### A residue in the FtsZ C-terminal conserved region is important for fast exchange in the Z-ring and recognition by ClpXP

**Fig 4:**
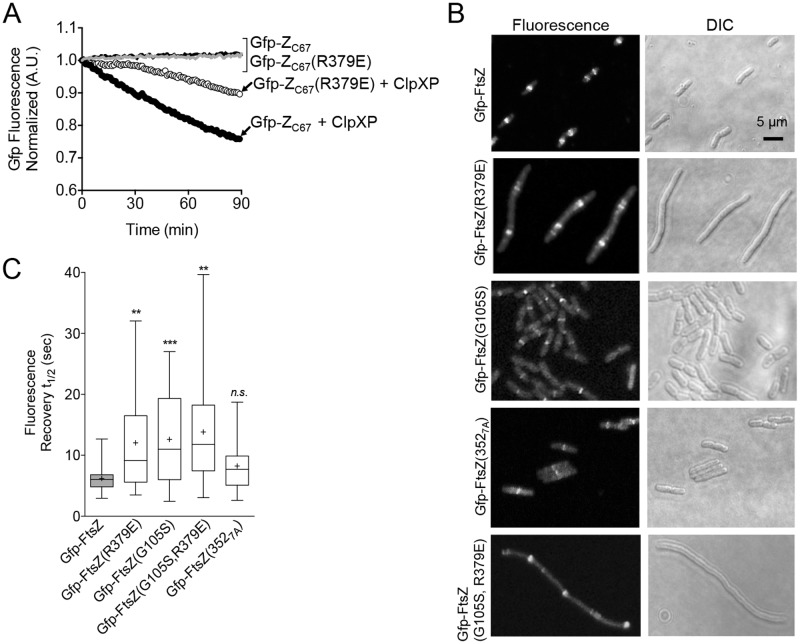
Mutation of ClpX interaction site impairs substrate degradation in vitro and dynamic exchange in vivo. (A) Degradation of Gfp-Z_C67_ (3 μM) and mutant Gfp-Z_C67_(R379E) (3 μM) was measured by monitoring loss of fluorescence with time in the presence (black and white circles, respectively) or absence (black and grey lines, respectively) of ClpXP (1 μM) where indicated, ATP (5 mM), and a regenerating system. The curves shown are representative of at least three replicates. (B) Fluorescence microscopy of wild type cells (JC0390) expressing Gfp-FtsZ, Gfp-FtsZ(R379E), Gfp-FtsZ(G105S), Gfp-FtsZ(352_7A_), or Gfp-FtsZ(G105S, R379E) induced with 140 μM arabinose under growth conditions described in *Materials and methods*. (C) Box and whiskers plot of average recovery half-times of Z-rings in wild type cells (JC0390) expressing Gfp-FtsZ, Gfp-FtsZ(R379E), Gfp-FtsZ(G105S), Gfp-FtsZ(352_7A_), or Gfp-FtsZ(G105S, R379E) induced with 140 μM arabinose under growth conditions described in *Materials and methods*. The extent of the box encompasses the interquartile range of the fluorescence recovery half-times, and whiskers extend to the maximum and minimum values. The line within each box represents the median, with the mean value indicated by ‘+’. Where indicated, *p* values are specified as ‘**’ (*p*<0.01), ‘***’ (*p*<0.001) or ‘n.s.’ (not significant) as compared to wild type.

The FtsZ mutant protein FtsZ(R379E) has a substitution at the end of the C-terminal conserved region and is defective for degradation by ClpXP in vitro [25]. To determine if Arg 379 is critical for targeting ClpX, we introduced the R379E substitution into Gfp-Z_C67_, and measured the degradation of purified Gfp-Z_C67_(R379E) by ClpXP in vitro by monitoring the loss of fluorescence with time. We observed that Gfp-Z_C67_(R379E) is degraded ~50% more slowly than Gfp-Z_C67_ indicating that Arg 379 is important for recognition and degradation by ClpXP (Fig 4A).

To investigate if FtsZ(R379E) is capable of assembly into functional Z-rings, we expressed Gfp-FtsZ(R379E) in the wild type parental strain (JC0390). Expression of Gfp-FtsZ(R379E) causes cells to be 2.7-fold longer than cells expressing Gfp-FtsZ (9.6 ± 0.6 μm and 3.5 ± 0.1 μm, respectively), and the cells have multiple Z-rings and additional fluorescent foci at sporadic locations (Fig 4B) (S4A Fig) (S1 Table). To investigate if the R379E substitution near the FtsZ C-terminus impairs the dynamic exchange of subunits in the Z-ring, we performed bleaching and recovery assays in wild type cells expressing Gfp-FtsZ(R379E) from a plasmid (S4B Fig). Expression of Gfp-FtsZ(R379E) in the wild type strain results in the assembly of a Z-ring that recovers fluorescence with a delayed average half-time of 12.1 ± 1.6 sec, which is 2-fold slower than Gfp-FtsZ (Fig 4C). Moreover, the individual half-time values are broadly distributed, similar to what we observed in *clp* deletion strains (Figs 4C and 3E) (S1 Table). These results suggest that Arg 379 in FtsZ is important for promoting fast exchange of FtsZ subunits in the Z-ring and is consistent with the role of ClpXP. Moreover, slow fluorescence recovery is not due to defective GTP hydrolysis or polymerization, since FtsZ(R379E) hydrolyzes GTP and assembles in vitro (S4E Fig) [25]. Furthermore, the expression levels of Gfp-FtsZ and Gfp-FtsZ(R379E) in vivo were similar for both strains (S4F Fig).

Mutation of G105S in FtsZ impairs GTP hydrolysis in vitro and confers a temperature-sensitive, filamentous phenotype in vivo; however, FtsZ(G105S) is capable of polymerization in vitro [14–16,51–53]. Cells expressing fluorescent Gfp-FtsZ(G105S) have previously been shown to exhibit slow fluorescence recovery after bleaching, even when grown at the permissive temperature, suggesting that the rate of GTP hydrolysis is coupled to subunit exchange in the Z-ring [12,13]. To confirm that impaired GTP hydrolysis also slows subunit exchange in vivo with the N-terminal Gfp-FtsZ fusion protein used in this study, we expressed Gfp-FtsZ(G105S) in the parental strain containing wild type *ftsZ* on the chromosome and measured fluorescence recovery after bleaching in the Z-ring (Fig 4B and 4C) (S4C Fig). We observed that Z-rings containing Gfp-FtsZ(G105S) recover fluorescence ~2-fold more slowly than cells expressing Gfp-FtsZ (12.6 ± 1.5 sec and 6.2 ± 0.5 sec, respectively) (Fig 4C) (S1 Table). Since mutation of either site (G105 or R379) is important, we tested if impairing both would further slow dynamic exchange in the Z-ring. We constructed Gfp-FtsZ(G105S, R379E) and visualized Z-rings in wild type cells. Gfp-FtsZ(G105S, R379E) localizes to Z-rings, but the cells are filamentous. We monitored fluorescence recovery in Z-rings but detected no further slowdown than observed for either mutation individually (Fig 4C) (S4D Fig) (S1 Table). We again observed a wide distribution of recovery times within the data set, compared to the parental strain expressing Gfp-FtsZ, and this heterogeneity may suggest a defect in regulating Z-ring assembly or maintenance. The expression levels of Gfp-FtsZ mutant proteins were similar to the level observed in cells expressing wild type Gfp-FtsZ (S4F Fig). As expected, we also observed that incorporation of the G105S mutation slows the rate of GTP hydrolysis of both FtsZ and FtsZ(R379E) using purified proteins in vitro (S4E Fig).

Next, we engineered alanine substitutions in the unstructured linker region of FtsZ spanning a second ClpX interaction site (residues 352–358) to construct FtsZ(352_7A_). We purified FtsZ(352_7A_) and confirmed that it hydrolyzes GTP and is partially defective for degradation by ClpXP in vitro (S4E and S5A Figs). We also constructed Gfp-FtsZ(352_7A_) and expressed it in dividing cells. We observed no obvious morphological defects and Z-rings appeared similar to cells expressing Gfp-FtsZ (Fig 4B). In bleaching and recovery assays of Z-rings containing Gfp-FtsZ(352_7A_), we observed a slightly wider distribution of recovery values than in rings containing Gfp-FtsZ, but the difference in average recovery half-time is not significant compared to Gfp-FtsZ (Fig 4C) (S5B Fig) (S1 Table).

### ClpX does not affect the dynamics of the Z-ring reporter ZipA-Gfp

**Fig 5:**
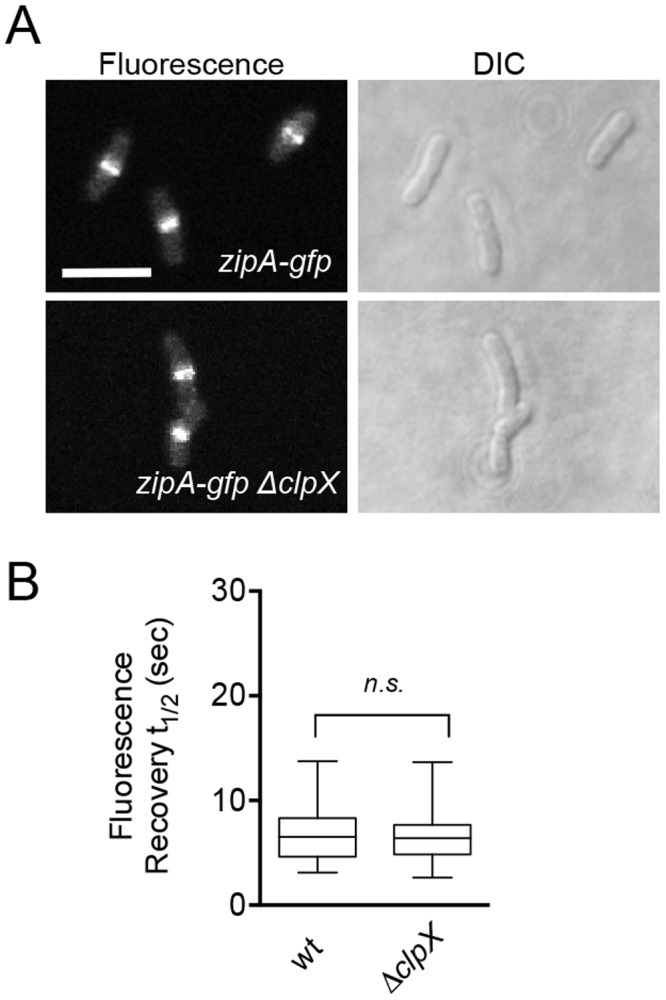
ZipA-Gfp ring assembly and dynamics are unaffected by deletion of *clpX*. (A) Fluorescence microscopy imaging of ZipA-Gfp rings in cells expressing ZipA-Gfp from the chromosome induced with 10 μM IPTG in strains with (MC181) and without *clpX* (MV0226) under growth conditions described in *Materials and methods*. Size bar is 2 μm. (B) Box and whiskers plot of average recovery half-times of ZipA-Gfp rings with (MC181) and without *clpX* (MV0226). The extent of the box encompasses the interquartile range of the fluorescence recovery half-times, and whiskers extend to the maximum and minimum values. The line within each box represents the median. Where indicated, *p* values are specified as ‘n.s.’ (not significant) as compared to wild type.

To determine if the effect that ClpXP has on fluorescence recovery time in the Z-ring is restricted to FtsZ or is observable using proteins that localize to a Z-ring after FtsZ localizes to the ring, we used cells expressing ZipA-Gfp from the chromosome and measured Z-ring dynamics in strains with and without *clpX* (Fig 5A) (Table 1). Midcell rings containing ZipA-Gfp are a marker for Z-ring formation and have a fast average recovery half-time, similar to the rate observed for Z-rings containing Gfp-FtsZ [13]. ZipA is not a putative substrate for ClpXP degradation and overproduction of ZipA has even been shown to protect FtsZ from degradation by ClpXP [54]. Upon expression of ZipA-Gfp, we observed fluorescent rings at midcell and cells appeared normal in length with and without chromosomally-encoded *clpX* under the conditions tested (MC181 and MV0226) (Fig 5A). We performed bleaching and fluorescence recovery assays on ZipA-Gfp rings in dividing cells with and without *clpX* and observed nearly identical average recovery half-times for ZipA-Gfp rings in both strains, 5.8 ± 0.6 sec in cells containing *clpX* and 5.8 ± 0.5 sec in cells deleted for *clpX* (Fig 5B) (S1 Table). Both ZipA-Gfp average recovery half-times are within error of Gfp-FtsZ in the wild type parental strain (JC0390) and unaffected by the presence or absence of ClpX (S1 Table).

### Cooperative degradation and disassembly of FtsZ polymers by ClpXP

**Fig 6:**
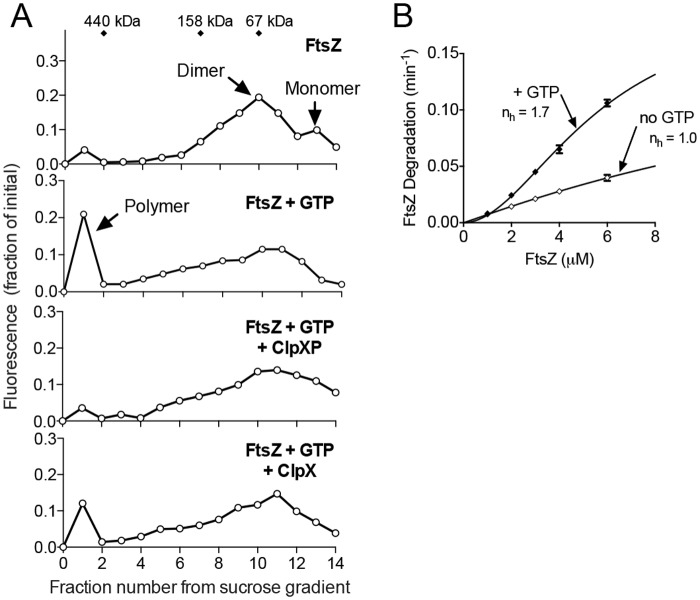
ClpXP degradation of FtsZ polymers. (A) Alexa Fluor labeled FtsZ (24 μM total) was polymerized with GTP (2 mM), where indicated, in the presence of a regenerating system, and then incubated alone, with ClpXP (1.6 μM) and ATP (5 mM) or with ClpX (1.6 μM) and ATP (5 mM). Reaction products were crosslinked with DSP, fractionated on a sucrose gradient and quantified by fluorescence. (B) Plot of the rate of degradation with increasing substrate concentration by ClpXP (1 μM) and Alexa Fluor labeled FtsZ (0 to 6 μM) with ATP (5 mM), a regenerating system and, where indicated GTP (2 mM). Hill coefficients (n_h_) were calculated by fitting the data to a nonlinear regression model as described in *Materials and methods*.

FtsZ polymers and unassembled FtsZ, which is a mixture of monomers and dimers, are degraded by ClpXP. In vitro, degradation reduces the overall abundance of polymers, and this is thought to occur as a result of degrading both populations, shifting the dynamic equilibrium of polymerized FtsZ in the direction of disassembly [24]. To determine if ClpXP reduces the size of existing FtsZ polymers, which would favor a directed disassembly model, we incubated ClpXP with FtsZ polymers, crosslinked the reaction products, and then analyzed their relative sizes by fractionation on a sucrose gradient. Using labeled fluorescent FtsZ (FtsZ-AF488) we observed that without GTP, FtsZ exists as a mixture of monomers (40.4 kDa) and dimers (80.8 kDa) (Fig 6A); however, when FtsZ was incubated with GTP and then crosslinked, we observed a large peak present at the high sucrose concentration (>440 kDa), corresponding to fractions 1 through 3, which together contained 25.1% of the total fluorescence in the initial reaction. We also detected fluorescence in the region between 158 kDa and 440 kDa, corresponding to fractions 4 through 6, which is consistent with short polymers and represents 14.5% of the total fluorescence (Fig 6A). In contrast, in the absence of GTP, minimal fluorescence was detected in regions corresponding to polymers (fraction 1 through 3) and short polymer fragments (fractions 4 through 6) amounting to 5.2% and 5.3% of the total fluorescence, respectively (Fig 6A). These results show that although FtsZ polymers formed in the presence of GTP are dynamic, incubation with dithiobis(succinimidyl propionate) (DSP) crosslinks a significant population of polymerized FtsZ, with the majority of polymers being very large. Next, we used this assay to test if incubation of FtsZ with ClpXP reduces the amount of large polymers detected. FtsZ polymers were assembled with GTP and then incubated with ClpXP prior to crosslinking. After incubation with ClpXP, we failed to detect a significant population of large polymers present in fractions 1 through 3 above the level of background (6.1% of total fluorescence) and even observed that there was less fluorescence in fractions 4 through 6, which corresponds to the region likely to contain short polymers, 10.1% of the total fluorescence with ClpXP, compared to 14.5% of the total fluorescence without ClpXP in fractions 4 through 6 (Fig 6A). Small molecular weight fluorescence was also detected in the reaction containing ClpXP in the low percent sucrose fraction due to the accumulation of fluorescent peptides, which are the products of degradation. However, the total fraction of fluorescent peptides was small, since FtsZ was in large excess over ClpXP. These results suggest that ClpXP disassembles large FtsZ polymers through degradation.

Finally, we used this assay to determine if ClpX alone, in the absence of ClpP, could also lead to fewer large FtsZ polymers, since ClpX has been reported to interact directly with FtsZ in vitro [36]. We observed a small reduction in the amount of fluorescence detected in polymer fractions 1 through 3 amounting to 15.4% of the total fluorescence with ClpX, compared to 25.1% of the total fluorescence without ClpX (but with GTP) in fractions 1 through 3 (Fig 6A). These results suggest that ClpX can modestly promote disassembly of large FtsZ polymers, but destabilization is more efficient in the presence of ClpP.

Interactions between FtsZ and several FtsZ-interacting proteins, including ZipA and SlmA, are mediated by multivalent interactions with FtsZ subunits within a polymer [42]. In addition, the rate of FtsZ degradation by ClpXP is enhanced when FtsZ is polymerized with GTP (Fig 1C) or the analog GMPCPP, as previously shown [24,25]. To determine if FtsZ polymers are recognized and degraded in a cooperative manner, we compared the rates of FtsZ degradation by ClpXP at concentrations above the critical concentration for FtsZ polymerization (1 μM) [2]. We observed that as the FtsZ concentration increased, the rate of degradation also increased in a positively cooperative manner (n_h_ = 1.7) when GTP was present (Fig 6B). In contrast, no cooperativity was observed when GTP was omitted from the degradation reactions (n_h_ = 1.0) and the overall rate of degradation was lower than in the presence of GTP. These results suggest that degradation of FtsZ polymers by ClpXP occurs by a cooperative recognition mechanism.

### Disassembly of FtsZ polymers is a mechanism to modulate Z-ring dynamics

**Fig 7:**
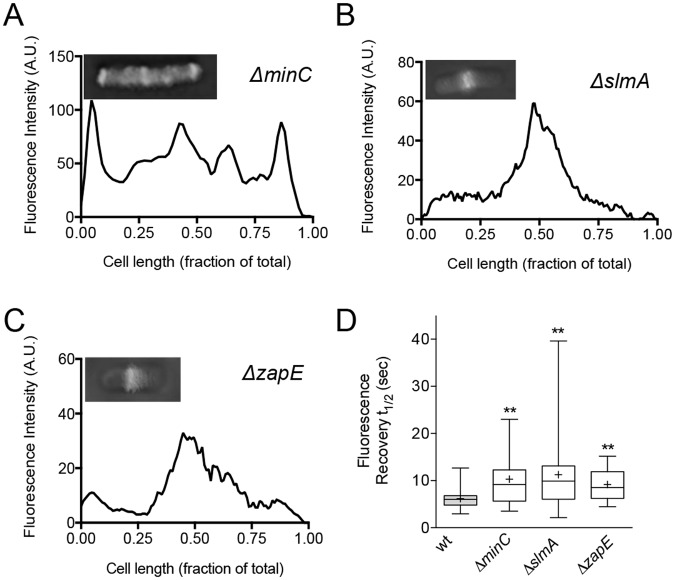
Slow Z-ring fluorescence recovery half-times in strains deleted for other cell division regulators. Fluorescence intensity across the long axis of the cell was measured and plotted for cells deleted for *minC* (JC0395) (A), *slmA* (MV0198) (B), and *zapE* (MV0277) (C) as described in *Materials and methods*. Inset shows the fluorescence image used for quantitation. Individual cells were chosen as representative of the population. (D) Plot for average fluorescence recovery half-times of bleached Z-rings in cells deleted for *minC* (JC0395), *slmA* (MV0198), and *zapE* (MV0277). The extent of the box encompasses the interquartile range of the fluorescence recovery half-times, and whiskers extend to the maximum and minimum values. The line within each box represents the median, with the mean value indicated by ‘+’. Where indicated, *p* values are specified as ‘**’ (*p*<0.01) as compared to wild type.

There are relatively few reports of factors that are capable of modifying Z-ring dynamics in *E*. *coli*. One previous report showed that deletion of the *minCDE* operon from *E*. *coli* slows Z-ring dynamics approximately 2-fold, similar to the effects observed here in *clp* deficient strains (Fig 3E) [12]. Since both MinC and ClpXP can destabilize FtsZ polymers in vitro, we investigated if destabilization of FtsZ polymers may be a mechanism to modulate Z-ring dynamics. Therefore, we expressed Gfp-FtsZ in strains deleted for *minC* (JC0395), *slmA* (MV0198), or *zapE* (MV0277), all of which are reported to promote FtsZ polymer disassembly, visualized fluorescent Z-rings and performed bleaching and recovery assays (Fig 7) (S6A and S6B Fig). In a *minC* deletion strain expressing Gfp-FtsZ, cells are mildly filamentous and contain multiple Z-rings (Fig 7A), which agrees with previous reports that deletion of *minC* leads to the formation of anucleate minicells as well as elongated cells with an increased number of Z-rings per cell [45,55–57]. Cells deleted for *slmA* or *zapE* show a single Z-ring present at the middle of the long-axis of the cell and are similar in length to the wild type strain (Fig 7B and 7C) (S1 Table). In all strains, approximately 20–30% of the total cellular fluorescence was present in a Z-ring (data not shown), similar to the wild type strain (Fig 1H). Next, we monitored fluorescence recovery of Z-rings in cells deleted for *minC*, *slmA*, or *zapE*. As expected, in cells deleted for *minC*, we observed a 70% longer average fluorescence recovery half-time than in the parental strain (Fig 7D) (S1 Table). Furthermore, reinsertion of *minC* back into the native locus by lambda-Red recombination restored MinC expression and rescued the slow Z-ring recovery of cells expressing Gfp-FtsZ to wild type half-times (S6A, S6C and S6D Fig) (S1 Table). We also observed slow Z-ring fluorescence recovery in the strain deleted for *slmA*, which showed a fluorescence recovery half-time that is on average 80% slower than in the wild type strain (Fig 7D) (S1 Table). Z-ring subunit exchange in cells deleted for *zapE* occurred faster than in the other deletion strains tested, but 50% slower than the parental strain (Fig 7D) (S1 Table). Taken together, our results suggest that proteins that function to destabilize FtsZ polymers in vitro enhance dynamic exchange in the Z-ring in vivo. Moreover, removal of *minC* and *slmA* widens the distribution of recovery values among the cells studied, indicating that there is increased heterogeneity throughout the population.

## Discussion

Using direct biochemical degradation assays, we demonstrate that an extended FtsZ C-terminal region (317–383), which includes two sites previously identified as important for ClpX recognition, is sufficient to target the non-native substrate Gfp for degradation (Fig 1B). We also show that full length FtsZ fused to Gfp at the N-terminus assembles into polymers with GTP and is also degraded by ClpXP in vitro (Fig 1C and 1D). As expected, degradation of Gfp-FtsZ occurs more rapidly in the presence of GTP, which promotes assembly of FtsZ into polymers. Titrating the FtsZ concentration above the critical concentration for polymer assembly shows a cooperative increase in the rate of degradation by ClpXP, which is consistent with multimerization enhancing recognition through concentration of FtsZ C-terminal binding sites (Fig 6B). In vivo Gfp-FtsZ localizes to the Z-ring, consistent with a previous report using a similarly constructed N-terminal Gfp-FtsZ fusion protein [58]. The polymerization domain is critical for FtsZ incorporation into the Z-ring because Gfp-Z_C67_ does not localize to a ring (data not shown), even though the protein interaction site near the C-terminus is intact.

The role of ClpXP during cell cycle progression in *C*. *crescentus* is well-characterized, but in *E*. *coli*, the functional role of ClpXP during division was unknown. Here we demonstrate that ClpXP is a regulator of Z-ring dynamics in vivo during division and that the regulation is proteolysis-dependent in vivo. In cells expressing Gfp-FtsZ, deletion of *clpX* or *clpP* leads to the formation of Z-rings that are on average ~70% slower to recover fluorescence after bleaching than Z-rings in the wild type parental strain. We also observed a large distribution of fluorescence recovery half-times, suggesting that although the cells appear normal, there is cell-to-cell variability in the stability of the Z-ring and the cells may be less resistant than wild type cells to further perturbations at the septa. Increased sensitivity may also explain why *clp* deletions are synthetic filamentous with a *minC* deletion [45]. By replacing the *clpP* gene with one that encodes a ClpP proteolytic mutant, ClpP(S97A), we confirmed that ClpXP degradation, not just ATP-dependent remodeling by ClpX, is critical for regulating division. Through direct degradation of FtsZ and the additional degradation of the Z-ring stabilizer ZapC, ClpXP likely has potent Z-ring destabilizing activity [59].

The dynamic nature of the Z-ring is widely reported, and other cell division proteins, including FtsA and ZipA, are known to localize to the dynamic ring [13,60]. How dynamics impact progression through the cell cycle is not well understood. Slow GTP hydrolysis by FtsZ is also linked to slow ring dynamics in vivo and was reported for Z-rings containing FtsZ(G105S) [13]. Constriction time could correlate with Z-ring dynamics. In a recent report by the Xiao group, deletion of *matP* was shown to shorten constriction time. Since MatP stabilizes the Z-ring through interactions with ZapA and ZapB, this suggests that FtsZ-interacting proteins modify the rate of constriction [17]. Interestingly, the dynamic ZipA-Gfp ring, often used as a marker for Z-ring assembly, is not modulated by ClpXP (Fig 5B). A recent study reported that although FtsZ and ZipA are thought to assemble early and form a proto-ring along with FtsA, FtsZ disassembles from the division septum prior to ZipA, suggesting that septal association of FtsZ and ZipA is differentially controlled [2,61–63]. The important role that FtsZ assembly regulators have in promoting division was recently demonstrated by showing that intragenic suppressor mutations in *ftsZ(G105S)* support division but rely on other cell division proteins, including ZapA [64].

Recognition of FtsZ by ClpX is complex and not yet fully understood. Although two sites in FtsZ are important for ClpXP degradation, the relative contributions of each site to degradation of distinct FtsZ conformations have yet to be determined. One may function as an auto-adaptor or enhancer, while the other may function as the degron, similar to the distinct sites of the ClpX substrate MuA [65]. Mutation of Arg 379 in native FtsZ or in Gfp-Z_C67_ impairs degradation by ClpXP in vitro (Fig 4A) [25]. Consistent with this, incorporation of Gfp-FtsZ(R379E) into Z-rings in vivo results in Z-rings that recover fluorescence more slowly than cells containing Gfp-FtsZ, suggesting that they are less dynamic due to defective degradation by ClpXP. The extreme C-terminus, also referred to as the C-terminal variable region, mediates bundling in other organisms such as *B*. *subtilis*, but not in *E*. *coli* [66]. We also investigated the second region of FtsZ in the unstructured linker that is involved in ClpX recognition, residues 352 through 358. Although FtsZ(352_7A_) is degraded more slowly than wild type FtsZ in vitro (S5A Fig), expression of Gfp-FtsZ(352_7A_) does not significantly perturb Z-ring assembly or fluorescence recovery in the Z-ring (Fig 4B and 4C) (S1 Table). Together, this suggests that the ClpX-interaction site near the FtsZ C-terminus is critical during division. Arg 379 may also be important for recognition by FtsA, which, along with ZipA, recruits FtsZ to the membrane in vivo [25,58,67–69]. Arg 379 is well conserved and also present in FtsZ from *Thermotoga maritima* [TmFtsZ(Arg344)] [70,71]. Accordingly, co-crystals of *T*. *maritima* FtsA and the C-terminal FtsZ peptide show that TmFtsZ(Arg344) forms a salt bridge with subdomain 2B of FtsA [70].

We also report that deletion of *minC*, *slmA* and *zapE* leads to Z-rings with slower dynamics. In a previous report, deletion of the *minCDE* operon was shown to slow the dynamic exchange in the Z-ring to a similar extent observed here by deletion of *minC* [12]. Notably, ZapA and ZapB, which stabilize FtsZ polymers in vitro, have been reported to stabilize the Z-ring in vivo [11,17]. It is therefore likely that a global function of FtsZ assembly modulators is to regulate the flux of subunits into and out of the Z-ring. In this way, the network of regulators ensures that appropriate exchange is maintained in the Z-ring during division.

## Materials and Methods

### Bacterial strains, plasmids, and growth conditions

*E*. *coli* strains and plasmids used in this study are described in Table 1. Strains were grown at 30°C in Lennox broth supplemented with appropriate antibiotics (kanamycin 50 μg ml^-1^, chloramphenicol 35 μg ml^-1^, and ampicillin 100 μg ml^−1^, where indicated). All MG1655 wild type and deletion strains used in bleaching and recovery assays contain constitutive promoter P_CP18_ in place of the chromosomal *araE* promoter, introduced by P1 transduction, to normalize cell-to-cell variation in expression [43]. Single gene kanamycin insertion-deletions were brought into MG1655 by P1 transduction using donor strains from the Keio collection [46]. For construction of histidine tagged Gfp-FtsZ and Gfp-Z_C67_, Gfp (Gfpuv) was cloned into pET28a(+) (EMD Millipore) as a *NheI*/*HindIII* fragment and the FtsZ extended C-terminal tail was cloned at an internal *SacI* site at the end of Gfp. FtsZ mutant proteins were constructed by site-directed mutagenesis of plasmids using the QuikChange II XL Site-Directed Mutagenesis Kit (Agilent).

To replace chromosomal *clpP* with *clpP(S97A)*, we first used site-directed mutagenesis of the ClpP expression plasmid (pET-ClpP) to construct pET-ClpP(S97A), and then amplified *clpP(S97A)* using recombination primers containing homology to 40-bp regions flanking the *clpP* locus. In the recipient strain, the *clpP* gene was deleted by lambda-Red recombination and replaced with a kanamycin cassette linked to the *parE* gene under the control of a rhamnose promoter ([50] and J. Teramoto, K. A. Datsenko, and B. L. Wanner, unpublished results). The amplified *clpP(S97A)* containing flanking sites for recombination was reinserted at the *clpP* locus and selected for by growth on L-rhamnose (1%). Recombinants were confirmed by sequencing. Similarly, *clpX*, *clpP* and *minC* wild type genes were restored in deletion strains at their native loci (Table 1).

### Expression and purification of proteins

ClpX, ClpP, FtsZ, and FtsZ(R379E) were each expressed in *E*. *coli* BL21 (λDE3) and purified as described [24,25,72,73]. FtsZ(G105S), FtsZ(G105S, R379E), and FtsZ(352_7A_) were purified as wild type FtsZ. FtsZ wild type and mutant proteins were labeled with Alexa Fluor 488 or 647, where indicated, and then fluorescent active subunits were obtained by cycles of polymerization and depolymerization [25,74]. Gfp-FtsZ and Gfp-Z_C67_ were overexpressed in *E*. *coli* BL21 (λDE3) grown in Lennox broth to an OD_600_ of 1.0 at 37°C and then induced with 1 mM IPTG for 3 hours at 30°C. Cells were lysed by French press, and soluble lysate was bound to TALON superflow resin (GE Healthcare). Histidine-tagged proteins were eluted with an imidazole gradient and imidazole was removed by buffer exchange. Protein concentrations are reported as FtsZ monomers, ClpX hexamers and ClpP tetradecamers.

### Degradation assays

Gfp-FtsZ and Gfp-Z_C67_ were degraded by ClpXP in buffer containing 20 mM HEPES pH 7.0, 150 mM KCl, and 10 mM MgCl_2_ with 5 mM ATP, 0.005% Triton X-100, and 2 mM GTP, where indicated, with acetate kinase (25 μg ml^-1^) and acetyl phosphate (15 mM) (bifunctional ATP/GTP regenerating system). Fluorescence was monitored with an Agilent Eclipse Spectrophotometer (excitation 395 nm, emission 510 nm). Degradation of FtsZ wild type and mutant proteins labeled with Alexa Fluor 488 or 647, where indicated, was performed as described by quantitating fluorescent peptides [25]. Hill coefficients were calculated by fitting the data to a nonlinear regression model using GraphPad Prism (version 6.0b) [Y = Rmax*X^h/(K^h + X^h), where Rmax is the maximum response, K is the concentration at half-maximal response and h is the Hill slope].

### Polymerization assays and GTP hydrolysis

Gfp-FtsZ and/or FtsZ was incubated with or without GTP (2 mM) for 3 min. in assembly buffer (50 mM MES, pH 6.5 100 mM KCl, 10 mM MgCl_2_) in the presence of a regenerating system (same one used for degradation assays) and spun for 30 min at 23°C in a Beckman TLA 120.1 rotor at 129,000*xg*. Supernatants and pellets were collected in equivalent volumes of 1x lithium dodecyl sulfate (LDS) sample buffer (Life Technologies) and analyzed by SDS-PAGE. GTP hydrolysis of FtsZ wild type and mutant proteins was assayed using the Biomol Green phosphate detection reagent (Enzo Life Sciences) as described [25].

### Sucrose gradient fractionation

Fluorescence-labeled FtsZ (24 μM) was incubated with and without GTP (2 mM), acetate kinase (25 μg ml^-1^) and acetyl phosphate (15 mM) in assembly buffer [50 mM MES (morpholino-ethane-sulfonic acid), pH 6.5, 100 mM KCl, 10 mM MgCl_2_] for 5 min. ClpXP (1.6 μM) and ATP (5 mM) were added, where indicated, and all reactions were incubated for 90 min. Reaction products were crosslinked with dithiobis(succinimidyl propionate) (DSP) (0.6 mM). After 30 min, reactions were quenched with Tris-HCl (25 mM, pH 8), applied to a 5–20% sucrose gradient with a 40% sucrose cushion, and centrifuged for 180 min at 4°C at 100,000*xg* in a Beckman TLS-55 rotor. Fractions (20 μl) were collected and analyzed by fluorescence.

### Microscopy

Overnight cultures of MG1655 wild type and deletion strains expressing Gfp-tagged (Gfpuv) FtsZ fusion proteins were diluted 1:50 into fresh media containing ampicillin (100 μg ml^−1^) and arabinose (70 μM or 140 μM, where indicated), and then grown for three hours at 30°C. Cells were directly applied to a 4% agarose pad containing MOPS [3-(*N*-morpholino) propanesulfonic acid] minimal media with 0.5% glycerol and a coverslip was added. Images were collected with a Zeiss LSM 700 confocal fluorescence microscope and images were captured on an AxioCam digital camera with ZEN 2012 software. In fluorescence recovery assays, regions of each Z-ring were selected and bleached at full laser power for one iteration until the initial fluorescence was reduced by at least 40%. Recovery images were captured at 3, 6 or 8 sec intervals, where indicated. The fluorescence intensity of the region at each interval was quantified using NIH ImageJ. Intensity values were normalized to the recovery period maximum plateau value, which was on average 70–80% of the initial pre-bleach fluorescence. Recovery was plotted as fluorescence intensity with time and fit to a nonlinear regression model {Y = Y0 + (Plateau-Y0)*[1-exp(-K*x)], where Y0 is the Y-value at time zero, Plateau is the Y value at infinite time and K is the rate constant} using GraphPad Prism (version 6.0b). Half-time recovery values were calculated for individual replicates and then averaged. The percentage of Z-ring fluorescence per cell was measured in ImageJ for at least 10 cells.

### Immunoblotting

Cells were grown as described and total proteins were precipitated with trichloroacetic acid (15% v/v). Proteins were resuspended in buffer containing 2% SDS, quantified by the bicinchoninic acid assay, and analyzed by SDS-PAGE and immunoblotting using antibodies to Gfp (Thermo Scientific), FtsZ, ClpX, ClpP or MinC as described [24,45].

## Supporting Information

S1 Fig ClpXP degradation of Gfp and ClpX unfolding of FtsZ chimeras in vitro. (A) Degradation of Gfp (3 μM) in the presence (black circles) and absence (white circles) of ClpXP (1 μM), ATP (5 mM) and a regenerating system was measured by monitoring loss of fluorescence with time. (B) Unfolding of Gfp-Z_C67_ (3 μM) in the presence of ClpX (1 μM), ATP (5 mM) and a regenerating system was measured by monitoring loss of fluorescence with time (grey circles). Unfolding of Gfp-FtsZ (5 μM) in the presence (green circles) and absence (yellow circles) of GTP, ClpX (1 μM), ATP (5 mM) and a regenerating system was measured by monitoring loss of fluorescence with time. For the unfolding of Gfp-FtsZ monomers in the absence of GTP, a regenerating system was used only for ATP containing creatine kinase (60 μg/ml) and phosphocreatine (5 mg/ml).
(PDF)

S2 Fig Fluorescence microscopy of Z-rings and replicate recovery curves in *clp* deficient strains. (A) Fluorescence microscopy of wild type cells (JC0390) expressing Gfp-FtsZ induced with 70 μM arabinose under growth conditions described in *Materials and methods* in cells deleted for *clpX* (JC0394), *clpP* (MV0210), with chromosomal *clpP(S97A)* (MV0256) in place of *clpP*, cells containing *clpP-restored* (MV03712) or *clpX-restored* (MV03722). Size bar is 2 μm. Replicate recovery curves for Z-rings containing Gfp-FtsZ in cells deleted for *clpX* (JC0394) (B), *clpP* (MV0210) (C), cells expressing chromosomal *clpP(S97A)* (MV0256) (D) in place of *clpP*, cells containing *clpP-restored* (MV03712) (E) or *clpX-restored* (MV03722) (F). Fluorescence recovery of each replicate was normalized to the maximal fluorescence observed during the recovery period and plotted with time. Immunoblot showing expression of ClpP (G) or ClpX (H) is restored in each deletion strain after replacement of the *parE-kan* cassette by lambda-Red recombination with *clpP* or *clpX* genes, where indicated.
(PDF)

S3 Fig Expression of Gfp-FtsZ at various arabinose concentrations and impact on Z-ring dynamics. (A) Immunoblot for Gfp-FtsZ in wild type cell (JC0390) extracts (1 μg of protein) expressing Gfp-FtsZ induced with 0, 70, or 140 μM arabinose under growth conditions for photobleaching experiments as described in *Materials and methods*. (B) Plot for average recovery half-times of Z-rings in wild type cells (JC0390) expressing Gfp-FtsZ induced with 70 or 140 μM arabinose under growth conditions for photobleaching experiments as described in *Materials and methods*.
(PDF)

S4 Fig Z-ring localization and fluorescence recovery in cells expressing Gfp-FtsZ(R379E) or Gfp-FtsZ(G105S). (A) Fluorescence intensity across the long axis of the cell was measured and plotted for a wild type cell (JC0390) expressing Gfp-FtsZ(R379E). The cell and plot shown are representative of the phenotype caused by expression of Gfp-FtsZ(R379E). Replicate half-time recovery curves for wild type cells (JC0390) expressing Gfp-FtsZ(R379E) (B), Gfp-FtsZ(G105S) (C), and Gfp-FtsZ(G105S, R379E) (D) induced with 140 μM arabinose under growth conditions for photobleaching experiments as described in *Materials and methods*. Fluorescence recovery of each replicate was normalized to the maximal fluorescence observed during the recovery period and plotted with time. (E) Rates of GTP hydrolysis for wild type FtsZ, FtsZ(R379E), FtsZ(G105S), FtsZ(G105S, R379E), and FtsZ(352_7A_). (F) Immunoblot for Gfp-FtsZ in wild type (JC0390) cells expressing pBad (empty vector), Gfp-FtsZ, Gfp-FtsZ(G105S), Gfp-FtsZ(R379E), Gfp-FtsZ(G105S, R379E), or Gfp-FtsZ(352_7A_) induced with 140 μM arabinose under growth conditions described in *Materials and methods* using antibodies to detect Gfp (1 μg of protein assayed).
(PDF)

S5 Fig Degradation of FtsZ(352_7A_) by ClpXP in vitro and fluorescence recovery of Gfp-FtsZ(352_7A_) in vivo. (A) Degradation reactions containing Alexa Fluor 647 labeled FtsZ(352_7A_) (5 μM total) in the presence of ClpXP (0.75 μM), ATP (5 mM), a regenerating system and GTP (2 mM), where indicated, were incubated for 30 minutes and then fluorescent degradation products were collected and quantified. (B) Replicate half-time recovery curves for wild type cells (JC0390) expressing Gfp-FtsZ(352_7A_) induced with 140 μM arabinose under growth conditions for photobleaching experiments as described in *Materials and methods*.
(PDF)

S6 Fig Fluorescence microscopy of Z-rings in cells deleted for other cell division proteins. (A) Fluorescence microscopy of Z-rings containing Gfp-FtsZ in wild type cells and cells deleted for *minC* (JC0395), *slmA* (MV0198), *zapE* (MV0277), and *minC-restored* (MV03732) under growth conditions for photobleaching experiments as described in *Materials and methods*. (B) Expression of Gfp-FtsZ in cell lysates (1 μg of protein) induced with 70 μM arabinose under growth conditions for photobleaching experiments described in *Materials and methods* for cells deleted for *minC* (JC0395), *clpX* (JC0394), *clpP* (MV0210), *slmA* (MV0198), and *zapE* (MV0277) using antibodies to detect Gfp (C) Replicate fluorescence recovery curves for Z-rings containing Gfp-FtsZ in *minC-restored* cells (MV03732). (D) Expression of MinC in *minC-restored* cells by immunoblot using antibodies to MinC.
(PDF)

S1 Table Cell lengths and fluorescence recovery times. (PDF)
