# Supplementary figures and images for: Proteolysis-Dependent Remodeling of the Tubulin Homolog FtsZ at the Division Septum in Escherichia coli
Source: PLoS One. 2017 Jan 23;12(1):e0170505. doi: 10.1371/journal.pone.0170505 (PMC5256927; doi:10.1371/journal.pone.0170505)

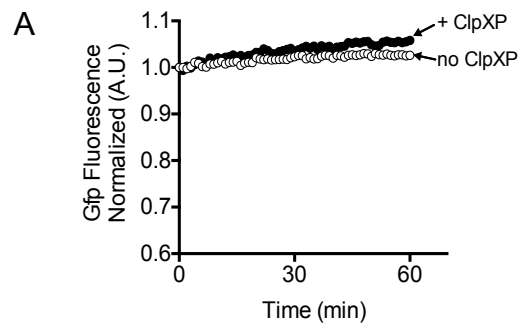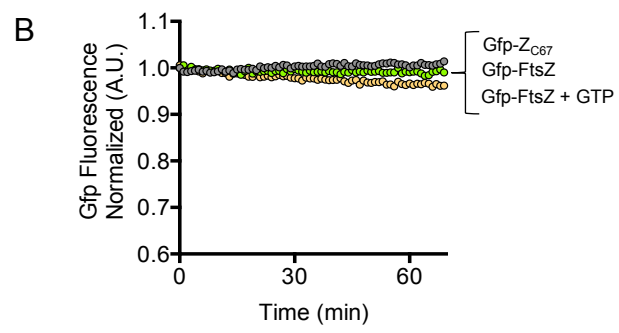

Supplement: S1 Fig — (A) Degradation of Gfp (3 μM) in the presence (black circles) and absence (white circles) of ClpXP (1 μM), ATP (5 mM) and a regenerating system was measured by monitoring loss of fluorescence with time. (B) Unfolding of Gfp-ZC67 (3 μM) in the presence of ClpX (1 μM), ATP (5 mM) and a regenerating system was measured by monitoring loss of fluorescence with time (grey circles). Unfolding of Gfp-FtsZ (5 μM) in the presence (green circles) and absence (yellow circles) of GTP, ClpX (1 μM), ATP (5 mM) and a regenerating system was measured by monitoring loss of fluorescence with time. For the unfolding of Gfp-FtsZ monomers in the absence of GTP, a regenerating system was used only for ATP containing creatine kinase (60 μg/ml) and phosphocreatine (5 mg/ml). (PDF) [file pone.0170505.s001.pdf]

A

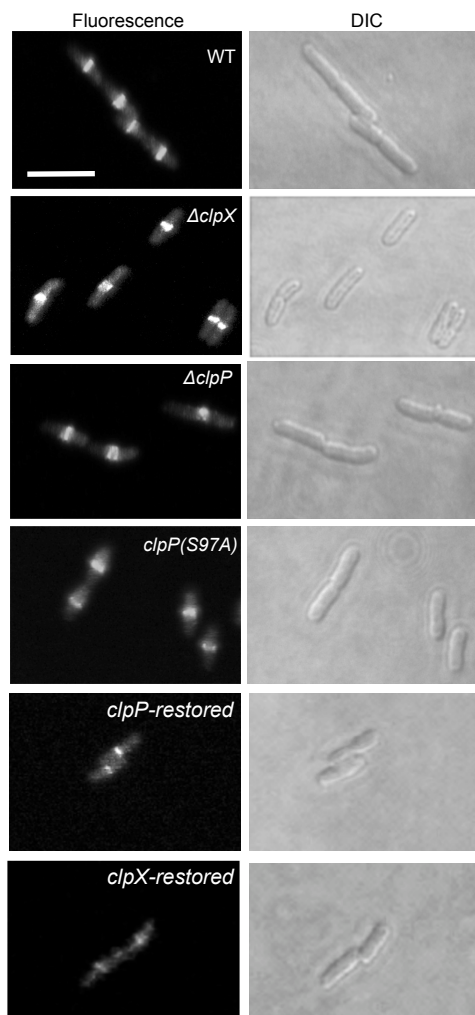

B

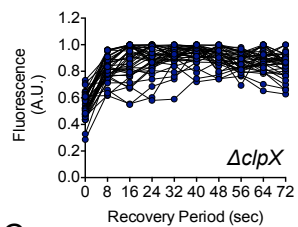

C

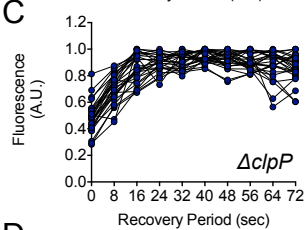

D

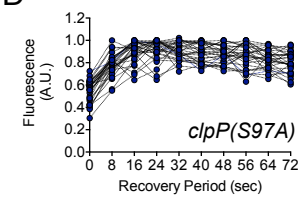

E

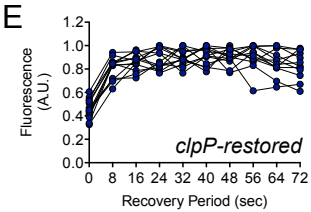

F

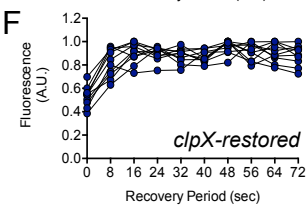

G

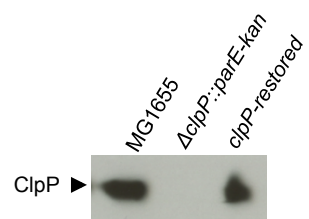

H

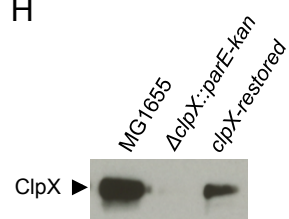

Supplement: S2 Fig — (A) Fluorescence microscopy of wild type cells (JC0390) expressing Gfp-FtsZ induced with 70 μM arabinose under growth conditions described in Materials and methods in cells deleted for clpX (JC0394), clpP (MV0210), with chromosomal clpP(S97A) (MV0256) in place of clpP, cells containing clpP-restored (MV03712) or clpX-restored (MV03722). Size bar is 2 μm. Replicate recovery curves for Z-rings containing Gfp-FtsZ in cells deleted for clpX (JC0394) (B), clpP (MV0210) (C), cells expressing chromosomal clpP(S97A) (MV0256) (D) in place of clpP, cells containing clpP-restored (MV03712) (E) or clpX-restored (MV03722) (F). Fluorescence recovery of each replicate was normalized to the maximal fluorescence observed during the recovery period and plotted with time. Immunoblot showing expression of ClpP (G) or ClpX (H) is restored in each deletion strain after replacement of the parE-kan cassette by lambda-Red recombination with clpP or clpX genes, where indicated. (PDF) [file pone.0170505.s002.pdf]

A

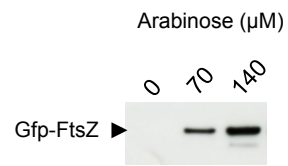

B

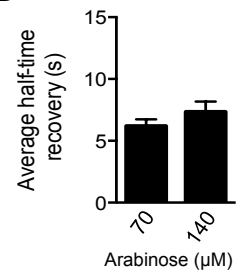

Supplement: S3 Fig — (A) Immunoblot for Gfp-FtsZ in wild type cell (JC0390) extracts (1 μg of protein) expressing Gfp-FtsZ induced with 0, 70, or 140 μM arabinose under growth conditions for photobleaching experiments as described in Materials and methods. (B) Plot for average recovery half-times of Z-rings in wild type cells (JC0390) expressing Gfp-FtsZ induced with 70 or 140 μM arabinose under growth conditions for photobleaching experiments as described in Materials and methods. (PDF) [file pone.0170505.s003.pdf]

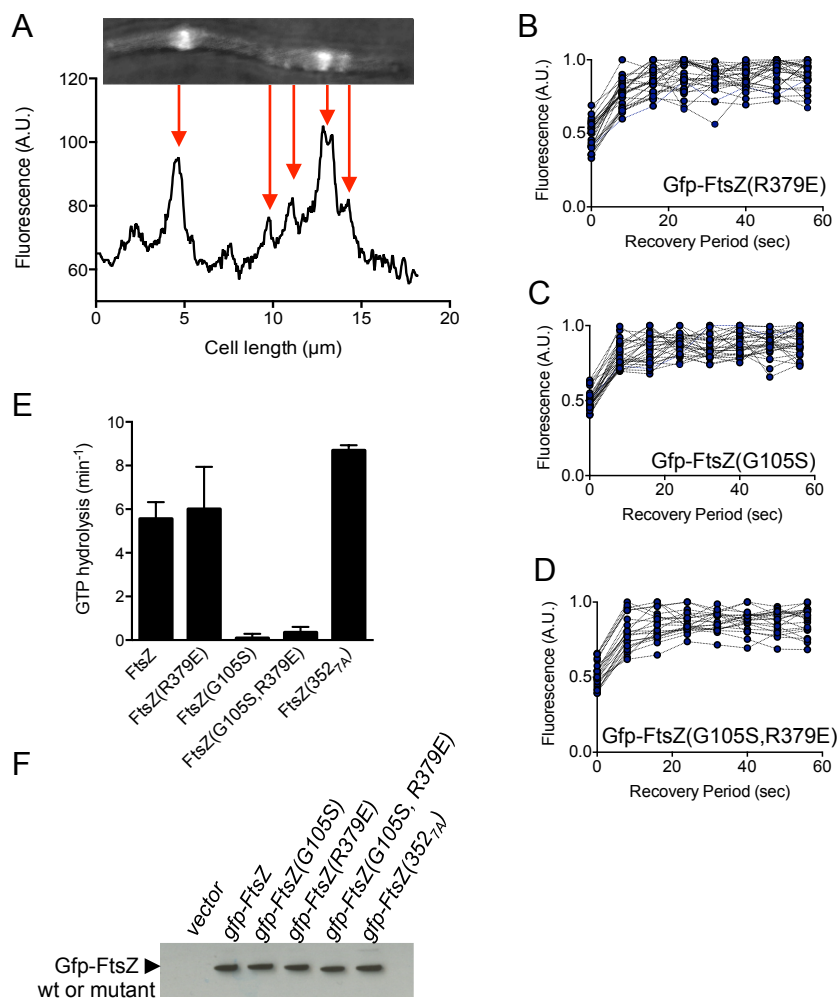

Supplement: S4 Fig — (A) Fluorescence intensity across the long axis of the cell was measured and plotted for a wild type cell (JC0390) expressing Gfp-FtsZ(R379E). The cell and plot shown are representative of the phenotype caused by expression of Gfp-FtsZ(R379E). Replicate half-time recovery curves for wild type cells (JC0390) expressing Gfp-FtsZ(R379E) (B), Gfp-FtsZ(G105S) (C), and Gfp-FtsZ(G105S, R379E) (D) induced with 140 μM arabinose under growth conditions for photobleaching experiments as described in Materials and methods. Fluorescence recovery of each replicate was normalized to the maximal fluorescence observed during the recovery period and plotted with time. (E) Rates of GTP hydrolysis for wild type FtsZ, FtsZ(R379E), FtsZ(G105S), FtsZ(G105S, R379E), and FtsZ(3527A). (F) Immunoblot for Gfp-FtsZ in wild type (JC0390) cells expressing pBad (empty vector), Gfp-FtsZ, Gfp-FtsZ(G105S), Gfp-FtsZ(R379E), Gfp-FtsZ(G105S, R379E), or Gfp-FtsZ(3527A) induced with 140 μM arabinose under growth conditions described in Materials and methods using antibodies to detect Gfp (1 μg of protein assayed). (PDF) [file pone.0170505.s004.pdf]

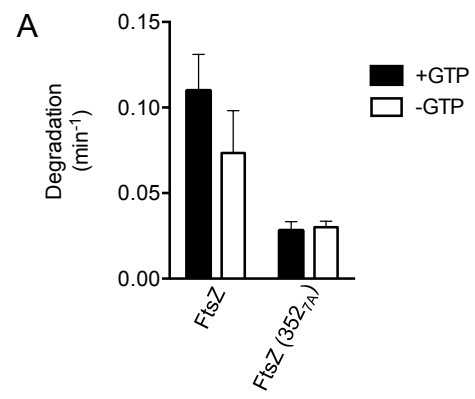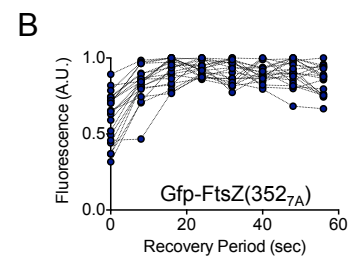

Supplement: S5 Fig — (A) Degradation reactions containing Alexa Fluor 647 labeled FtsZ(3527A) (5 μM total) in the presence of ClpXP (0.75 μM), ATP (5 mM), a regenerating system and GTP (2 mM), where indicated, were incubated for 30 minutes and then fluorescent degradation products were collected and quantified. (B) Replicate half-time recovery curves for wild type cells (JC0390) expressing Gfp-FtsZ(3527A) induced with 140 μM arabinose under growth conditions for photobleaching experiments as described in Materials and methods. (PDF) [file pone.0170505.s005.pdf]

A

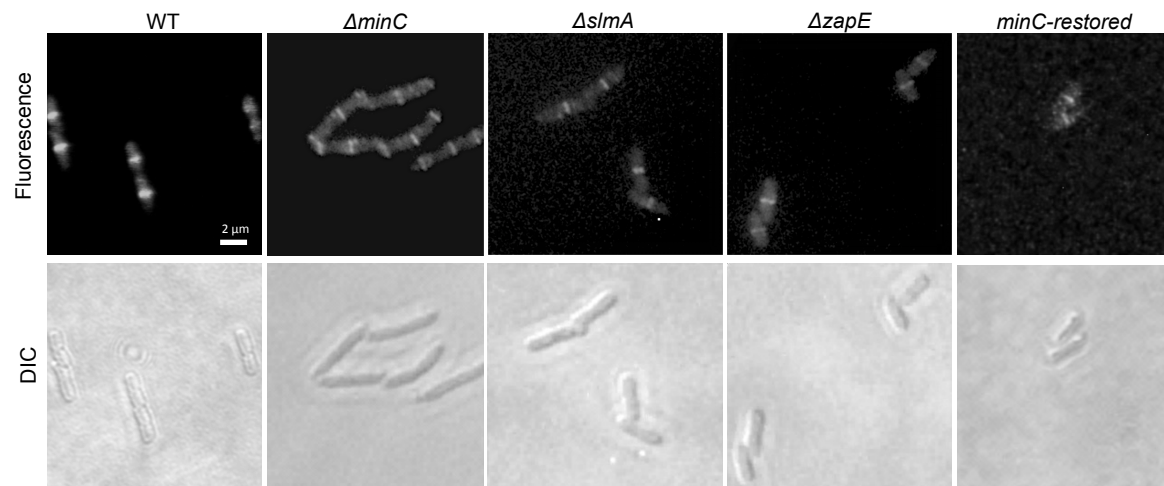

B

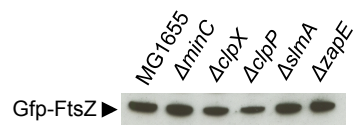

C

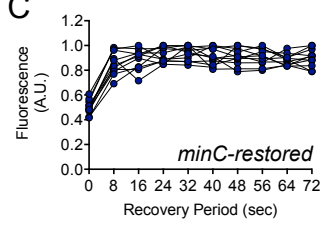

D

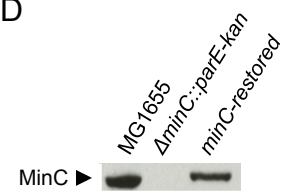

Supplement: S6 Fig — (A) Fluorescence microscopy of Z-rings containing Gfp-FtsZ in wild type cells and cells deleted for minC (JC0395), slmA (MV0198), zapE (MV0277), and minC-restored (MV03732) under growth conditions for photobleaching experiments as described in Materials and methods. (B) Expression of Gfp-FtsZ in cell lysates (1 μg of protein) induced with 70 μM arabinose under growth conditions for photobleaching experiments described in Materials and methods for cells deleted for minC (JC0395), clpX (JC0394), clpP (MV0210), slmA (MV0198), and zapE (MV0277) using antibodies to detect Gfp (C) Replicate fluorescence recovery curves for Z-rings containing Gfp-FtsZ in minC-restored cells (MV03732). (D) Expression of MinC in minC-restored cells by immunoblot using antibodies to MinC. (PDF) [file pone.0170505.s006.pdf]
