# Supplementary material for: Proteolysis-Dependent Remodeling of the Tubulin Homolog FtsZ at the Division Septum in Escherichia coli
Source: PLoS One. 2017 Jan 23;12(1):e0170505. doi: 10.1371/journal.pone.0170505 (PMC5256927; doi:10.1371/journal.pone.0170505)
Supplement: S1 Table — (PDF) [file pone.0170505.s007.pdf]

Strain or plasmid	Relevant Genotype	Average cell length[a] (µm)	Fluor. Recovery Average $t_{1/2}$ (sec)[b]	*p* value[c]
**Host Strain + pGfp-FtsZ**				
JC0390	*wild type*	3.5 ± 0.1	6.2 ± 0.5 (n=18)	-
JC0394	*ΔclpX*	3.1 ± 0.1	10.6 ± 1.3 (n=38)	0.0037
MV0210	*ΔclpP*	3.0 ± 0.1	10.6 ± 0.7 (n=27)	<0.0001
MV0256	*clpP(S97A)*	2.7 ± 0.04	10.4 ± 1.4 (n=30)	0.0072
JC0395	*ΔminC*	6.1 ± 0.2	10.3 ± 1.2 (n=20)	0.0049
MV0198	*ΔslmA*	4.4 ± 0.2	11.2 ± 1.7 (n=22)	0.0091
MV0277	*ΔzapE*	4.3 ± 0.1	9.2 ± 0.8 (n=18)	0.0048
MV03722	*clpX-restored*	n.d.	8.2 ± 1.4 (n=8)	n.s.
MV03712	*clpP-restored*	n.d.	8.1 ± 0.6 (n=11)	n.s.
MV03732	*minC-restored*	n.d.	6.7 ± 1.1 (n=9)	n.s.
**MG1655 (JC0390) + plasmid**				
pGfp-FtsZ(G105S)		4.0 ± 0.1	12.6 ± 1.5 (n=25)	0.0003
pGfp-FtsZ(R379E)		9.6 ± 0.6	12.1 ± 1.6 (n=26)	0.0013
pGfp-FtsZ(352_{7A})		n.d.	8.2 ± 1.1 (n=18)	n.s.
pGfp-FtsZ(G105S, R379E)		> 10 µm	13.8 ± 2.3 (n=18)	0.0041
**BW27784 *zipA-gfp***				
MC181		5.4 ± 0.1	5.8 ± 0.6 (n=21)	
MV0226	*ΔclpX*	4.3 ± 0.1	5.8 ± 0.5 (n=23)	

[a] Average cell length was calculated by measuring a minimum of 200 cells; 'n.d.' is not determined.

[b] Error values are reported as SEM.

[c] *p* values for each data set compared to wild type strain JC0390 expressing Gfp-FtsZ were calculated by Welch's t test . A *p* value >0.01 was not significant (n.s.).
